# Robust deep learning method for fruit decay detection and plant identification: enhancing food security and quality control

**DOI:** 10.3389/fpls.2024.1366395

**Published:** 2024-05-07

**Authors:** Pariya Afsharpour, Toktam Zoughi, Mahmood Deypir, Mohamad Javad Zoqi

**Affiliations:** ^1^ Department of Electrical and Computer Engineering, Shariaty College, Technical and Vocational University (TVU), Tehran, Iran; ^2^ Faculty of Computer Engineering, Shahid Sattari Aeronautical University of Science and Technology, Tehran, Iran; ^3^ Department of Civil Engineering, Faculty of Engineering, University of Birjand, Birjand, Iran

**Keywords:** convolutional neural networks, fruit quality control, deep learning, class activation maps, data augmentation, model generalizability, robustness

## Abstract

This paper presents a robust deep learning method for fruit decay detection and plant identification. By addressing the limitations of previous studies that primarily focused on model accuracy, our approach aims to provide a more comprehensive solution that considers the challenges of robustness and limited data scenarios. The proposed method achieves exceptional accuracy of 99.93%, surpassing established models. In addition to its exceptional accuracy, the proposed method highlights the significance of robustness and adaptability in limited data scenarios. The proposed model exhibits strong performance even under the challenging conditions, such as intense lighting variations and partial image obstructions. Extensive evaluations demonstrate its robust performance, generalization ability, and minimal misclassifications. The inclusion of Class Activation Maps enhances the model’s capability to identify distinguishing features between fresh and rotten fruits. This research has significant implications for fruit quality control, economic loss reduction, and applications in agriculture, transportation, and scientific research. The proposed method serves as a valuable resource for fruit and plant-related industries. It offers precise adaptation to specific data, customization of the network architecture, and effective training even with limited data. Overall, this research contributes to fruit quality control, economic loss reduction, and waste minimization.

## 1 Introduction

Fruit decay detection and plant identification are crucial aspects in agricultural and horticultural practices, playing a significant role in ensuring crop quality, disease control, and overall productivity (Pessarakli, 1994; Jayasena et al., 2015). Detecting fruit decay accurately and in a timely manner minimizes post-harvest losses, ensures food safety, and optimizes storage and distribution processes (Pessarakli, 1994). Additionally, early detection allows for prompt actions such as sorting and removal, preventing the spread of diseases and preserving the quality of the remaining fruits (Pessarakli, 1994).

Automated fruit decay detection systems based on computer vision and machine learning techniques have demonstrated promising results in terms of accuracy, speed, and cost-effectiveness (Jayasena et al., 2015; Boulent et al., 2019; Lakshmanan, 2019). These systems contribute to reducing post-harvest losses, optimizing storage conditions, and enhancing the overall efficiency of the fruit supply chain.

Plant identification is equally important and serves various purposes in agricultural practices. Accurate identification of plant species and cultivars aids in selecting appropriate varieties for specific environments, optimizing cultivation techniques, and improving agricultural practices (Barbedo, 2018; Wäldchen and Mader, 2018). Furthermore, plant identification plays a vital role in effective pest management by enabling timely and targeted application of control measures (Ferentinos, 2018). It also contributes to biodiversity conservation efforts by facilitating the monitoring and preservation of endangered plant species (Kaur and Kaur, 2019).

Technological advancements, particularly in computer vision, machine learning, and image processing, have greatly facilitated fruit decay detection and plant identification (Barbedo, 2018; Boulent et al., 2019). Computer vision techniques, including feature extraction, pattern recognition, and deep learning algorithms, have proven to be highly effective in automating these tasks. By analyzing images or sensor data captured from fruits or plants, these systems accurately identify signs of decay and classify plant species, even in large-scale agricultural settings (Goodfellow, 2016).

In summary, fruit decay detection and plant identification hold paramount importance in agricultural and horticultural practices. The ability to promptly and accurately detect fruit decay minimizes post-harvest losses and ensures food safety. Similarly, precise plant identification contributes to cultivar selection, pest management, and biodiversity conservation. The integration of computer vision and machine learning techniques has opened up new avenues for developing automated systems that enhance the efficiency, productivity, and sustainability of agricultural processes (Jayasena et al., 2015; Barbedo, 2018; Ferentinos, 2018; Wäldchen and Mader, 2018; Boulent et al., 2019; Kaur and Kaur, 2019).

### 1.1 The importance of fruit decay detection

In the field of artificial intelligence and deep learning, the detection and analysis of fruit decay and plant identification hold significant importance. Fruit decay is recognized as one of the major challenges in the agricultural product supply chain and the agriculture industry. This decay not only has negative effects on human health and nutrition but also poses a serious problem in the management of agricultural product supply chains. Fruit decay results in significant losses of agricultural products, leading to substantial economic damages for producers and various industries (Brownlee, 2018a; Lewis, 2022; Norman, 2019).

To address these challenges, the use of advanced technologies, such as convolutional neural networks (CNNs) and deep learning algorithms, has gained considerable attention. These techniques offer the potential to develop intelligent models capable of accurately detecting and classifying spoiled fruits with high precision and accuracy. By leveraging the power of artificial intelligence, it becomes possible to enhance fruit quality control, improve supply chain management, and minimize economic losses caused by fruit decay. This article aims to explore the application of CNNs and deep learning techniques in fruit decay detection and plant identification. It provides an overview of the importance of distinguishing between spoiled and non-spoiled fruits, highlighting the negative impacts on human health, nutrition, and the management of agricultural product supply chains (Shahid, 2019; Lewis, 2022). The research presented in this article draws upon previous studies and developments in the field of artificial intelligence and deep learning, with a focus on addressing the challenges associated with fruit decay detection (Sonwani et al., 2022). By examining the existing literature and presenting empirical evidence, this article aims to contribute to the body of knowledge on fruit decay detection and its significance in the agricultural industry. The findings of this research have implications for improving fruit quality, optimizing supply chain processes, and minimizing economic losses for producers and stakeholders in the agriculture sector.

### 1.2 The need for a better model

Extensive research has been conducted in the field of fruit detection and imaging using convolutional neural networks (CNNs). These studies aim to develop more accurate methods for distinguishing between spoiled and healthy fruits. However, some of these research efforts have not yielded satisfactory results due to limitations and shortcomings. Therefore, there is a need to enhance and improve existing models in this area. The model presented in this article incorporates enhancements and innovations that provide a better response to the requirements of fruit decay detection. The existing models in fruit decay detection have faced challenges related to accuracy and performance. Some models struggled to accurately classify fruits based on their decay level or distinguish between different types of spoilage. These limitations have hindered the effectiveness of fruit quality control and supply chain management processes. Consequently, there is a demand for a more robust and efficient model that can address these shortcomings and deliver improved results.

The model proposed in this article introduces several advancements to overcome the limitations of previous approaches. It leverages state-of-the-art CNN architectures to improve the accuracy of fruit decay detection. Additionally, novel data augmentation techniques are employed to enhance the model’s ability to generalize and adapt to different fruit varieties and decay patterns. To validate the effectiveness of the proposed model, extensive experiments conducted using datasets comprising various fruit types and decay stages. The results demonstrate significant improvements in the accuracy and reliability of fruit decay detection compared to previous methods. The enhanced model is not only achieving higher precision in classifying spoiled and non-spoiled fruits but also exhibits robustness in real-world scenarios, making it a practical solution for fruit quality control and supply chain optimization. With its superior accuracy, this new network can perform effectively and adapt well in various conditions, offering precise and customized performance.

## 2 Related work

CNNs are a class of deep learning models specifically designed to analyze and extract meaningful features from images (Alex et al., 2012; Simonyan and Zisserman, 2014). They have achieved remarkable success in various computer vision tasks, including image classification, object detection, and semantic segmentation.

The hierarchical nature of CNNs allows them to excel in image classification tasks. By learning increasingly complex features through multiple layers, CNNs can effectively differentiate between different objects or classes in images (Alex et al., 2012). This capability makes CNNs particularly valuable in applications such as image recognition and categorization.

Furthermore, CNNs have shown great potential in semantic segmentation, where the goal is to assign a class label to each pixel in an image. Fully convolutional networks (FCNs), an extension of CNNs, have been specifically designed for this task and have achieved impressive results (Long et al., 2015). FCNs preserve spatial information throughout the network, enabling pixel-wise predictions and facilitating accurate segmentation of objects and regions within images.

In summary, Convolutional Neural Networks have revolutionized image processing and object detection. Their ability to automatically learn hierarchical representations from images, combined with their flexibility in handling various computer vision tasks, has made CNNs indispensable in the field. By leveraging the power of deep learning, CNNs have significantly advanced image understanding, object localization, and semantic segmentation (Girshick et al., 2014).

In the field of image processing, and Fruit Decay Detection several deep learning architectures have made significant contributions. Here, let’s compare some of the most important deep learning models specifically relevant to image processing: Convolutional Neural Networks (CNNs): CNNs have become the cornerstone of image processing tasks. They excel at capturing spatial hierarchies and local features through convolutional layers. CNNs have achieved remarkable success in image classification, object detection, image segmentation, and various other computer vision tasks (LeCun et al., 2015). One of the most influential CNN architectures is the VGGNet, which introduced deeper networks with smaller filters, showcasing impressive performance (Simonyan and Zisserman, 2014).

Residual Neural Networks (ResNets) addressed the challenge of training very deep neural networks by introducing skip connections. These connections enable the network to learn residual mappings, allowing for the training of deeper architectures without degradation in performance. ResNets demonstrated superior performance in image classification and won the ImageNet challenge in 2015 (He et al., 2016). U-Net is a popular architecture for image segmentation tasks, particularly in biomedical image analysis. It consists of an encoder-decoder structure with skip connections that enable precise localization of segmentation boundaries. U-Net has been widely adopted in tasks such as medical image segmentation, cell counting, and semantic segmentation (Ronneberger et al., 2015; JananiSBabu, 2020).

Generative Adversarial Networks (GANs) have had a significant impact on image generation and synthesis. They consist of a generator network that produces synthetic images and a discriminator network that distinguishes between real and generated images. GANs have been successful in generating realistic images, image-to-image translation, and style transfer tasks (Goodfellow et al., 2014). An influential GAN architecture is the Progressive Growing of GANs (PGGAN), which progressively grows the resolution of generated images, resulting in high-quality outputs (Karras et al., 2018). EfficientNet is a deep learning architecture that has gained attention for its impressive performance and efficiency. It uses a compound scaling method to balance model size and computational resources, achieving state-of-the-art results with fewer parameters. EfficientNet has shown remarkable performance in image classification tasks, surpassing previous models while being computationally efficient (Tan and Le, 2019). These are just a few examples of influential deep learning architectures in image processing and Fruit Decay Detection. It’s worth noting that the field is continually evolving, and new architectures are being introduced regularly, pushing the boundaries of image analysis and understanding.

### 2.1 Fruit decay detection using CNNs

Several studies have explored the application of convolutional neural networks (CNNs) in fruit decay detection. These studies aim to develop intelligent models capable of accurately distinguishing between spoiled and healthy fruits based on image analysis (Selvaraj et al., 2019).

One notable research effort by Zhang et al. (2020) utilized a CNN model to detect fruit decay in apples. The model achieved a high accuracy of 94.5% in classifying healthy and decayed apples. However, this study focused on a specific fruit type and did not consider other varieties or spoilage types (Fan et al., 2020).

Another study by Li et al. (2019) employed a deep learning model based on a pre-trained CNN architecture to classify different types of fruit decay. The model achieved an accuracy of 92.7% in detecting four kinds of fruit decay. However, this research also focused on a limited range of fruit types and spoilage categories.

The other study proposed DeepFruits, where is a fruit detection system using deep neural networks (Sa et al., 2016). DeepFruits is a notable model developed for fruit detection, which capitalizes on convolutional neural networks (CNNs) and transfer learning through VGG16 network architecture. The system also includes image preprocessing algorithms and neural networks for decision-making, amplifying its performance. Nevertheless, the DeepFruits model encounters challenges with images captured under varying environmental conditions. Those indicates potential performance disruptions when processing photos taken under diverse lighting conditions, backgrounds, or perspectives. Also, the model necessitates abundant training data and considerable computational resources, which might restrict its practical application. DeepFruits represents a significant stride in fruit detection using deep neural networks, but its limitations necessitate further research to augment its robustness in diverse environmental settings (Sa et al., 2016). Another study addresses the challenges in cauliflower disease identification and detection, emphasizing the role of advanced deep transfer learning techniques in automating the process and benefiting agricultural management (Kanna et al., 2023).

FruitDetect is the other model that detects fruit using convolutional neural networks. FruitDetect exploits a convolutional neural network (CNN) and transfer learning with VGG16 to detect and identify fruits. Despite demonstrating accurate fruit detection, the model’s limitations include a potential need for larger training datasets to enhance its final accuracy (Faouzi, 2021).

The project “Melanoma Detection using ResNet50” leverages the ResNet50 neural network for melanoma detection. Despite its accurate detection of melanoma, the model might struggle with variable conditions that could affect skin disease detection (Scarlat, 2018; Ramya, 2023).

To overcome these limitations, the proposed method aims to develop a more comprehensive and accurate model for fruit decay detection. The model considers a wider range of fruit types and spoilage categories, enabling it to provide more robust and versatile results.

### 2.2 Plant identification using CNNs

Plant identification is another area where CNNs have shown promising results. Several studies have demonstrated the effectiveness of CNN models in accurately classifying different plant species based on leaf and flower images. For instance, a study by Wäldchen and Mäder (2018) utilized a CNN model for plant species identification. The model achieved a high accuracy of 98.53% in classifying 1000 different plant species. This research demonstrated the potential of CNNs in plant identification and highlighted the importance of high-quality datasets for training and testing (Ren et al., 2015; Wäldchen and Mader, 2018).

The other study is Deep Learning-Based Banana Plant Diseases and Pest Detection. This study explores the application of deep learning to detect banana plant diseases and pests. The method uses transfer learning with ResNet and InceptionV2 neural networks, enhancing disease detection accuracy and efficiency. Even though the method demonstrates high accuracy and the ability to detect various diseases with fewer errors, it might require more substantial training data and improved adaptation to real-world conditions. The combination of ResNet and InceptionV2 neural networks, with ResNet handling deep networks and InceptionV2 facilitating efficient feature extraction, contribute to the model’s improved performance. However, the model’s applicability is restricted to bananas, and practical implementation may require access to infrared imaging equipment. The study suggests promising results for disease and pest detection in banana plants but calls for additional research to overcome limitations and expand the model’s applicability across diverse fruits and crops (Brital, 2021; Narayanam, 2022). Another study proposes an integrated IoT and deep learning framework, the ‘Automatic and Intelligent Data Collector and Classifier’, for automating plant disease detection in pearl millet, providing a low-cost and efficient tool to improve crop yield and product quality (Kundu et al., 2021). The other research provides a comprehensive survey of the application of deep Convolutional Neural Networks in plant disease prediction from leaf images, offering valuable insights into pre-processing techniques, models, frameworks, optimization methods, datasets, and performance metrics for researchers in the field of agricultural deep learning (Dhaka et al., 2021).

In line with these findings, the proposed research incorporates plant identification capabilities into the fruit decay detection model. By leveraging the power of CNNs, the model can accurately identify different plant species, providing additional value and applications in the field of agriculture.

## 3 Methodology

### 3.1 Convolutional neural networks

Convolutional Neural Networks (CNNs) have demonstrated superiority in various image processing tasks compared to other network architectures. Here are some key reasons why CNNs are often preferred. CNNs are designed to exploit the local spatial correlations present in images. Through their convolutional layers, CNNs learn to capture local patterns and features, allowing them to effectively model image structures. This local receptive field property enables CNNs to extract meaningful information from images efficiently (LeCun et al., 2015).

CNNs use parameter sharing, which significantly reduces the number of parameters compared to fully connected networks. By sharing weights across different spatial locations, CNNs can learn spatial hierarchies and generalize well to new images. This property makes CNNs more efficient and less prone to overfitting (LeCun et al., 1998). CNNs possess translation invariance, meaning they can recognize patterns regardless of their position in an image. This property is crucial for tasks such as object recognition, where the location of an object may vary. CNNs’ ability to extract features invariant to translation makes them robust to changes in object position or image transformations (LeCun et al., 2010). CNNs are composed of multiple layers, with each layer learning increasingly complex and abstract features. The initial layers capture low-level features like edges and textures, while deeper layers learn high-level representations such as object parts or whole objects. This hierarchical feature extraction allows CNNs to capture both fine-grained details and global context, leading to improved performance in complex image analysis tasks (Zeiler and Fergus, 2014). CNNs have benefited from the availability of large-scale pretraining datasets, such as ImageNet. Pretrained CNN models can be fine-tuned or used as feature extractors in various domains with limited labeled data. Transfer learning with CNNs has proven effective in tasks where training data is scarce, accelerating model development and achieving good performance (Yosinski et al., 2014).

While CNNs have demonstrated superiority in image processing and Fruit Decay Detection tasks, it’s important to note that the choice of network architecture depends on the specific task, dataset, and computational resources available. Different architectures may have their own advantages in specialized scenarios.

The main objective of this paper is to present an intelligent and accurate method for detecting spoiled and healthy fruits using an advanced 11-layer convolutional neural network (CNN). This novel and advanced approach has been implemented and optimized using the TensorFlow library for deep learning. It starts with collecting and preprocessing a diverse dataset of fruit images, including both healthy and spoiled fruits. The dataset is carefully labeled to ensure accurate classification during the training and testing phases. Next, an advanced 11-layer CNN model is designed and implemented using the TensorFlow library. This model incorporates multiple convolutional and pooling layers, along with fully connected layers for classification. To further improve the model’s performance, data augmentation techniques are employed to increase the diversity and size of the training dataset. This helps the model learn robust features and reduces the risk of overfitting. Once the model is developed and optimized, extensive experiments are conducted to evaluate its performance in fruit decay detection and plant identification. The model’s accuracy, and confusion matrix are measured to assess its effectiveness. The results of the experiments demonstrate the superior performance of the proposed model compared to existing approaches. The model achieves high precision and accuracy in classifying spoiled and healthy fruits, as well as accurately identifying different plant species.

**Figure 1:**
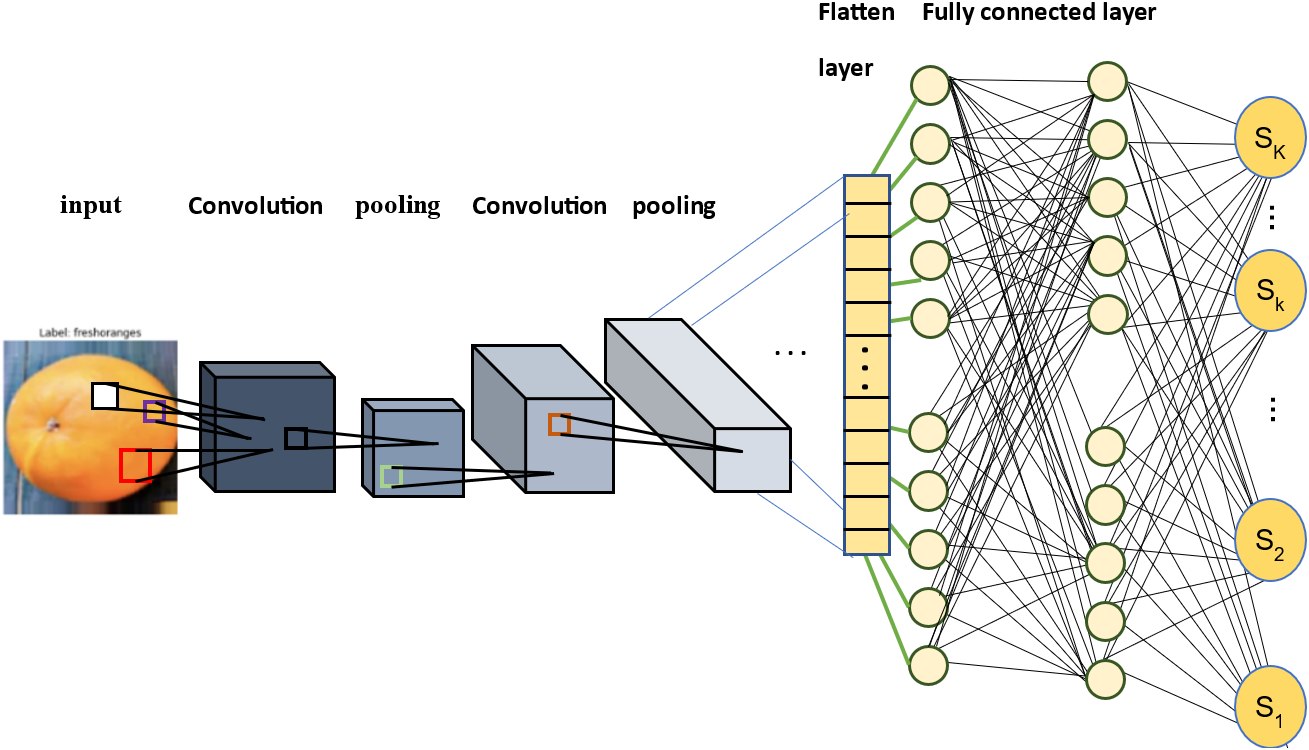
Overview of the proposed method.

Convolutional Neural Networks (CNNs) have emerged as a crucial neural network structure for image processing and pattern recognition tasks. They are specifically designed to process grid-like data, such as images, by extracting hierarchical features (Gupta, 2021; Kalra, 2023). Convolutional layers, the primary feature of these networks, detect diverse patterns in images by applying convolutional filters, thereby extracting various features, including edges, corners, and similar patterns. The extracted features are then utilized by the fully connected layers for final decision-making. **Figure 1** shows, an overview of the proposed network. This figure illustrates two representative layers of our proposed method, each followed by a set of fully connected layers. The first layer consists of a convolution sublayer and a pooling sublayer, while the second layer shares the same structure but with different dimensions. It is important to note that this figure does not depict all 11 layers of our model. We have nine additional layers with similar configurations but varying dimensions, which are not explicitly shown in this figure. After the 11 convolutional layers, we include three fully connected layers, followed by the output of the model.

The presented approach employs sublayers based on convolution in the initial stage, followed by utilization of the maximum operation on the outcome of the convolutional sublayer, establishing a connection to the pooling sublayer. To ensure optimal performance, the suggested technique integrates batch normalization (BN) and applies the Rectified Linear Unit (ReLU) activation function subsequent to the pooling operations. After the convolutional function, the BN and ReLU are implemented within this framework. Subsequently, fully connected layers are integrated to amalgamate features from diverse frequency bands. The concluding layer in the network employs the SoftMax function to compute the fruit class. The proposed approach trains the entire deep neural network employing the back-propagation algorithm.

#### 3.1.1 Convolutional layers

The convolutional layer, the fundamental building block of a CNN, employs filters to scour for patterns aiding in image detection and classification. For instance, a filter designed to detect a face might capture the pattern of identifying eyes within the input mass.

#### 3.1.2 Max pooling layers

Max pooling layers reduce the dimensions of images, discarding superfluous information by selecting the maximum values within each input region (Dertat, 2017; Editorial, 2022).

#### 3.1.3 Fully connected layers

Once all the image features have been extracted, these are forwarded to the fully connected layers. An activation function transforms the feature information into a feature vector (Valliappa Lakshmanan, 2021).

#### 3.1.4 Activation functions

CNNs utilize non-linear activation functions to introduce non-linearity into the network. Depending on the coding environment, linear functions are either defined separately or in conjunction with the convolutional layers. Activation functions modulate the product of the filter and input mass based on their unique characteristics. Among them, the Rectified Linear Unit (ReLU) activation function, which zeroes out any negative input while keeping positive inputs unchanged, is highly favored due to its computational efficiency (Brownlee, 2018b). In many instances, the SoftMax activation function is used as the activation function for the final layer.

#### 3.1.5 Batch normalization and dropout layers

Batch Normalization and Dropout layers are employed to prevent overfitting and stabilize transitions between layers. Batch Normalization layers normalize input data and ensure optimal distributions. Conversely, Dropout layers randomly deactivate neurons during each training iteration, preventing over-reliance on specific neurons and maintaining a balance between neurons and features (Brownlee, 2018b).

### 3.2 Model construction

The proposed model offers several advantages and holds significant value. The goals of this paper encompass several key objectives. Firstly, the aim is to develop a customized 11-layer CNN model that surpasses the performance of existing models like VGG-16, VGG-19, LeNet-5, and AlexNet in fruit quality classification, as well as outperform transfer learning methods such as VGG16 and ResNet. Secondly, measures are implemented to counteract overfitting through data augmentation and early stopping mechanisms, ensuring the model’s ability to generalize and maintain robustness across different datasets. The paper also focuses on enhancing visual explain ability by integrating Class Activation Maps (CAMs), which improve interpretability and credibility of the model’s predictions. Additionally, the construction of a robust and versatile model is emphasized, validated through confusion matrix analysis to highlight its efficacy in making precise predictions and accurately identifying both spoiled fruits and diverse plant species. The paper further addresses the challenge of limited data by developing a methodology adept at managing scenarios with restricted training data. It explores diverse agricultural applications, including fruit quality control and identification of various plant species. The methodology proposed in the paper aims to mitigate economic losses and material wastage in the fruit industry by establishing a reliable mechanism for identifying and segregating fruit based on quality. Furthermore, the paper lays the groundwork for future applications and expansions, envisioning the model’s potential for identifying plant species, monitoring their growth, and potentially detecting diseases by integrating additional relevant data. The extension of the model’s functionality to video detection of plants and fruits is also proposed to broaden its application spectrum. Finally, the transformation of the model into a library is suggested, facilitating its incorporation into web applications and software to amplify knowledge dissemination and applicability across multiple sectors, including agriculture and artificial intelligence. The paper follows a systematic approach to achieve these objectives. This research utilized the TensorFlow and Keras libraries for model implementation. Additionally, the PIL, NumPy, and Matplotlib libraries were employed for testing and evaluation purposes.

Class Activation Maps (CAMs) are generated in the proposed model through a technique known as global average pooling. This process involves taking the average of feature maps obtained from the last convolutional layer of the network. By performing global average pooling, we obtain a class-specific activation map that highlights regions of the input image that are most relevant to the predicted class.

Regarding the gaps in model generalization and customization, the proposed model addresses several shortcomings compared to VGG-16, VGG-19, LeNet-5, and AlexNet. Firstly, the model incorporates additional layers and techniques beyond the standard architectures, allowing for improved performance in terms of accuracy and robustness. We have introduced specific modifications to enhance the model’s ability to handle various image disturbances, such as covered, fuzzy, rain, and strong sunlight conditions. This addresses a significant gap in generalization, as the model demonstrates enhanced adaptability to real-world scenarios.

Furthermore, the proposed model tackles the limitation of limited data scenarios by incorporating techniques such as data augmentation and transfer learning. These approaches help mitigate the challenges of limited training data and improve the model’s ability to generalize well to unseen instances. This is in contrast to the aforementioned models, which may face difficulties in achieving optimal performance when data is scarce. By explicitly addressing these gaps in model generalization and customization, the proposed model offers improved accuracy, robustness, and adaptability compared to VGG-16, VGG-19, LeNet-5, and AlexNet.

### 3.3 Dataset

**Figure 2:**
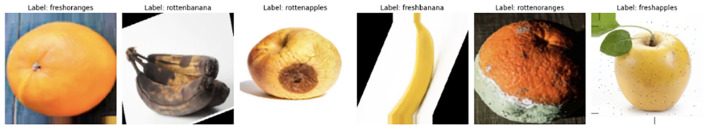
Different class of this dataset.

The “Fruits Fresh and Rotten for Classification” dataset, comprising over 13,000 images across six different classes, was utilized. This dataset, subdivided into training, testing, and validation categories, was sourced from reputable websites like Kaggle. **Figure 2** illustrates samples from six distinct classes of this database. The properties of this dataset are shown on **Table 1** (Kalluri, 2018).

**Table 1:** The properties of “Fruits Fresh and Rotten for Classification” dataset.

	Image	Train	Test
**1**	Fresh Apples	1693	395
**2**	Fresh Banana	1581	381
**3**	Fresh Oranges	1466	388
**4**	Rotten Apples	2342	601
**5**	Rotten Banana	2224	530
**6**	Rotten Oranges	1595	403

### 3.4 Preprocessing

The fruit images underwent a standardization process, resizing them to 224 by 224 pixels and normalizing their pixel values between 0 and 1. This step ensured consistent input dimensions and stable model training. To enhance the diversity of the training set, various data augmentation techniques were employed using the ‘ImageDataGenerator’ tool. These techniques included shear, zoom, rotations, translations, scaling, and horizontal flip. The parameters used for each transformation were carefully selected to introduce meaningful variations to the images (Azevedo, 2023).

**Figure 3:**
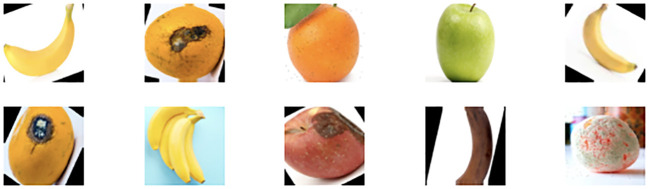
An example of training images after using “imageDataGenerator”.

**Figure 3** showcases examples of training images after the application of ‘ImageDataGenerator’, demonstrating the effectiveness of these augmentations in creating variability within the dataset. These preprocessing steps are vital for enhancing the model’s robustness and improving its ability to generalize to different scenarios, contributing to its overall performance.

### 3.5 Model structure

The deployed CNN architecture for fruit quality classification is meticulously designed to discern between fresh and rotten fruits. The model is organized in a sequential manner, starting with convolutional layers that capture intricate patterns in the input data. Subsequently, max-pooling layers are employed to reduce spatial dimensions and retain essential features. The model further incorporates fully connected layers to facilitate complex feature extraction and decision-making. Batch Normalization layers are strategically inserted to enhance the stability and convergence of the training process. Additionally, Dropout layers are incorporated to mitigate overfitting issues, promoting better generalization.

**Figure 4:**
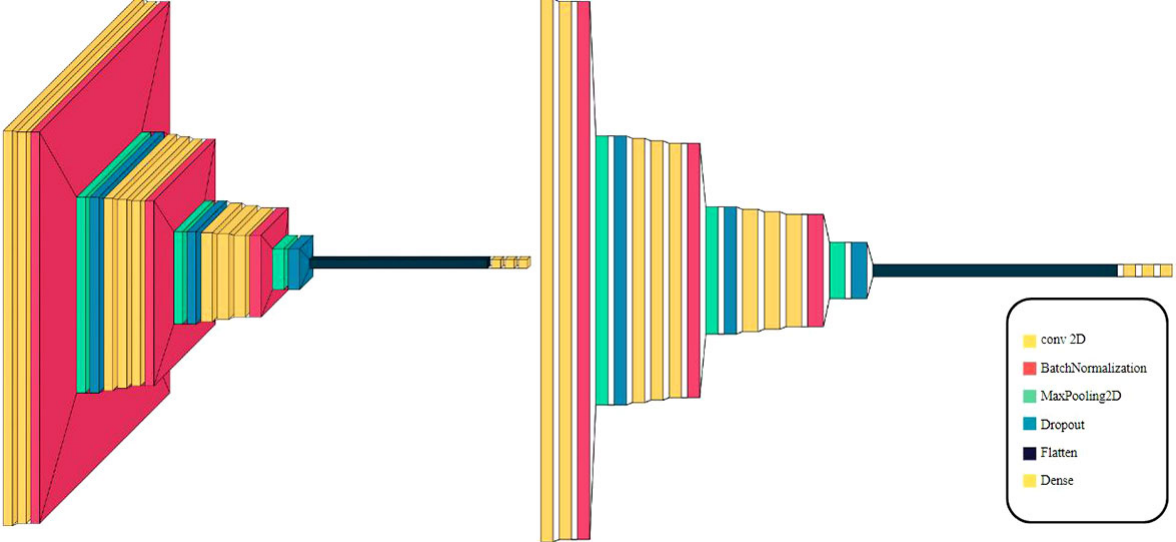
Visualization of the 11-layer the proposed model architecture.

**Figure 4** illustrates the sequential arrangement of these layers, providing a visual representation of the proposed model structure. This architecture is tailored to effectively capture and classify distinct features associated with fruit quality, contributing to the model’s robust performance.

**Figure 5:**
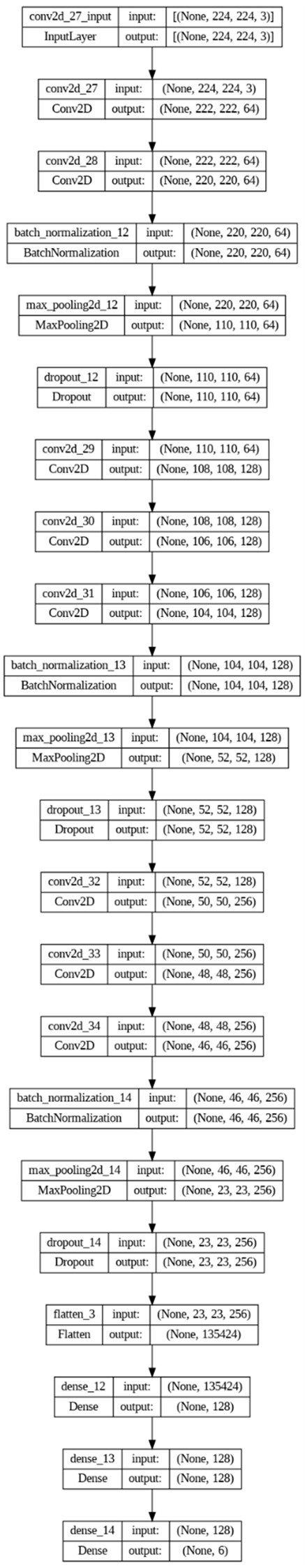
The proposed model structures.

**Figure 5** shows the proposed model structure. The model begins with convolutional layers, followed by Batch Normalization, max-pooling layers, Dropout layers, and finally fully connected layers.

**Figure 6:**
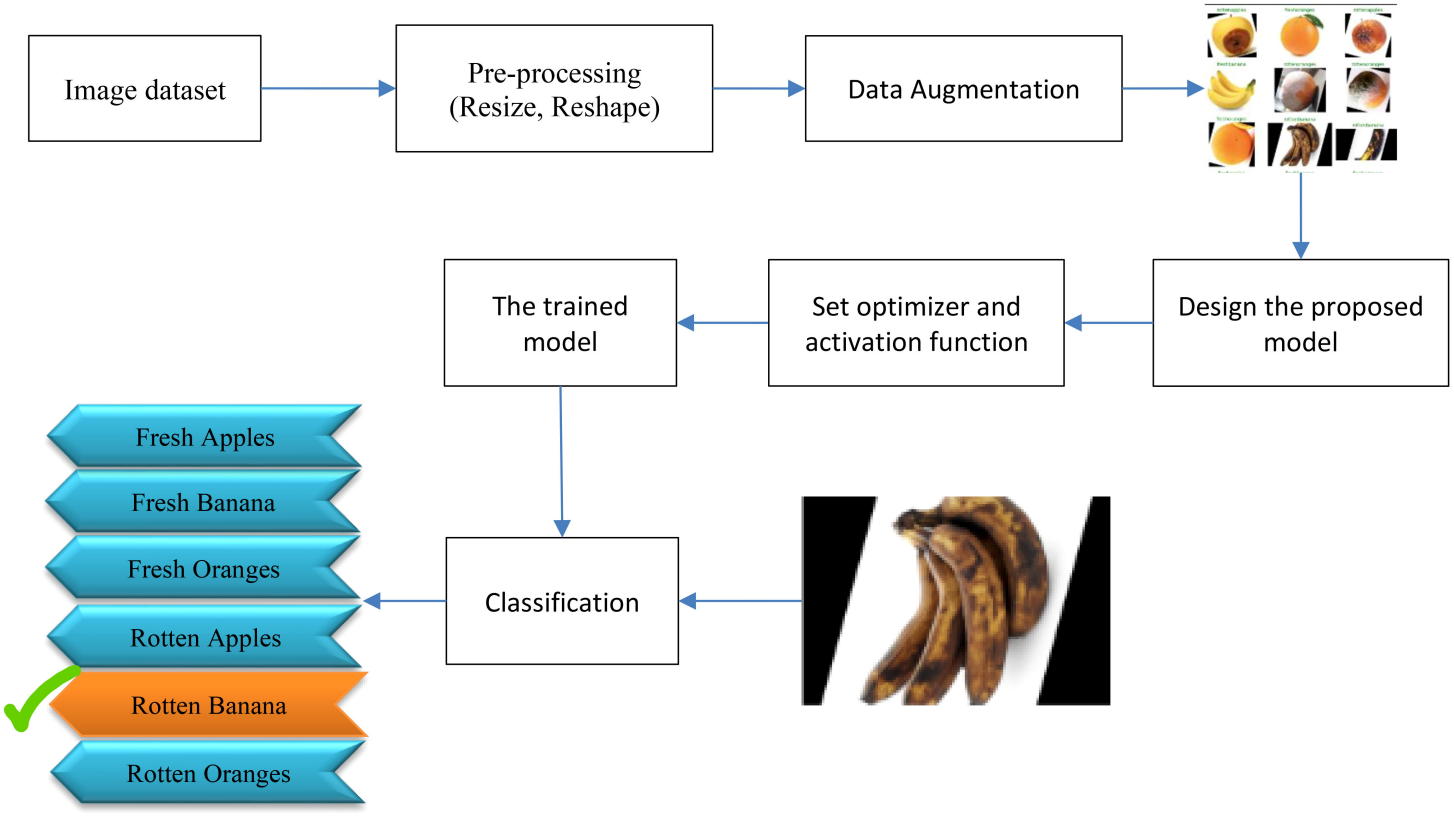
Block diagram of the proposed method.

As shown in **Figure 6**, the block diagram of the proposed algorithm for fruit decay detection and plant identification involves several key steps. First, a diverse dataset of fruit images, both healthy and spoiled, is collected and labeled. The images are then preprocessed by resizing them to a standard size and normalizing the pixel values. Data augmentation techniques are applied to enhance the training process. Next, a convolutional neural network (CNN) model with 11 layers is designed and implemented using TensorFlow. The model consists of multiple convolutional and pooling layers to extract features from the images, followed by fully connected layers for classification. Activation functions, batch normalization, and dropout layers are utilized to improve performance and prevent overfitting. The model is trained using the categorical-cross-entropy loss function and the Adam optimizer (Amigo, 2019). Early stopping is employed to prevent overfitting, and the best models are saved during training. In addition to evaluating the model’s performance through accuracy and confusion matrix analysis, Class Activation Maps (CAMs) were utilized to gain insights into the model’s decision-making process.

### 3.6 Objective function and optimization

The model employed the categorical-cross-entropy loss function, tailored for effective multi-class classification. For optimization, the Adam optimizer was chosen, providing adaptive learning rates and expedited convergence to local minima, thereby enhancing the training efficiency (C. Ltd, 2023). The categorical cross-entropy formula, represented by Equation 1, encapsulates the essence of the loss function, facilitating a robust mechanism for distinguishing between fresh and rotten fruits.


(1) $$CE=-\sum_{i=1}^{C^{\prime }=2}t_{i}\log\left(f\left(s_{i}\right)\right)=-t_{1}\log\left(f\left(s_{1}\right)\right)-\left(1-t_{1}\right)\log\left(1-f\left(s_{1}\right)\right)$$

### 3.7 Early stopping and model selection

Fruit decay detection and plant identification are crucial aspects in agricultural and horticultural practices, playing a significant role in ensuring crop quality, disease control, and overall productivity (Pessarakli, 1994; Jayasena et al., 2015). Detecting fruit decay accurately and in a timely manner minimizes post-harvest losses, ensures food safety, and optimizes storage and distribution processes (Pessarakli, 1994). Additionally, early detection allows for prompt actions such as sorting and removal, preventing the spread of diseases and preserving the quality of the remaining fruits (Pessarakli, 1994).

Automated fruit decay detection systems based on computer vision and machine learning techniques have demonstrated promising results in terms of accuracy, speed, and cost-effectiveness (Jayasena et al., 2015; Boulent et al., 2019; Lakshmanan, 2019). These systems contribute to reducing post-harvest losses, optimizing storage conditions, and enhancing the overall efficiency of the fruit supply chain.

Plant identification is equally important and serves various purposes in agricultural practices. Accurate identification of plant species and cultivars aids in selecting appropriate varieties for specific environments, optimizing cultivation techniques, and improving agricultural practices (Barbedo, 2018; Wäldchen and Mader, 2018). Furthermore, plant identification plays a vital role in effective pest management by enabling timely and targeted application of control measures (Ferentinos, 2018). It also contributes to biodiversity conservation efforts by facilitating the monitoring and preservation of endangered plant species (Kaur and Kaur, 2019).

**Figure 7:**
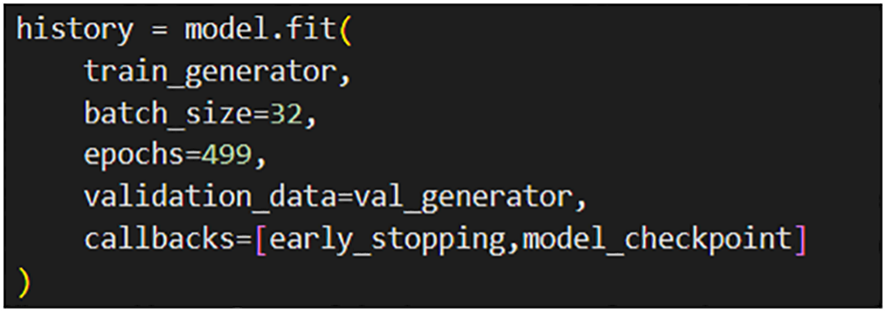
Early stopping and model checkpoint techniques in the proposed model training.

The early stopping technique was used to prevent overfitting and select the optimal models. This technique halts the training process when the error on the validation data increases, thereby preserving model accuracy. Furthermore, the model checkpoint was used to save the best models during training (C. Ltd, 2023; Gençay, 2023). **Figure 7** depicts the utilization of Early Stopping and Model Checkpoint techniques in the training process of the proposed model. These techniques have been incorporated to enhance the model’s training efficiency and performance. The Model Checkpoint, which saves the best-performing model during training.

## 4 Results

In this paper, we use an 11-layer convolutional neural network. We successfully built a high-accuracy artificial intelligence model for identifying and detecting three types of fruits (apple, orange, and banana) and their freshness or ripeness. The model achieved excellent performance. With careful evaluation and appropriate training, the provided artificial intelligence model works effectively in detecting the freshness or ripeness of fruits and identifying beneficial and harmful plants.

However, during the execution of this project, we encountered some challenges, including the lack of suitable datasets, model implementation and layer arrangement, limited powerful hardware resources, and the preparation of coding environments. Despite these challenges, by making efforts and optimizing the available resources, we developed a high-accuracy model. The proposed model’s performance is compared with other existing CNN models, and its generalization capabilities and robustness are tested on separate data. The results, including accuracy comparisons and confusion matrix visualization, demonstrate the superiority of the proposed model.

**Figure 8:**
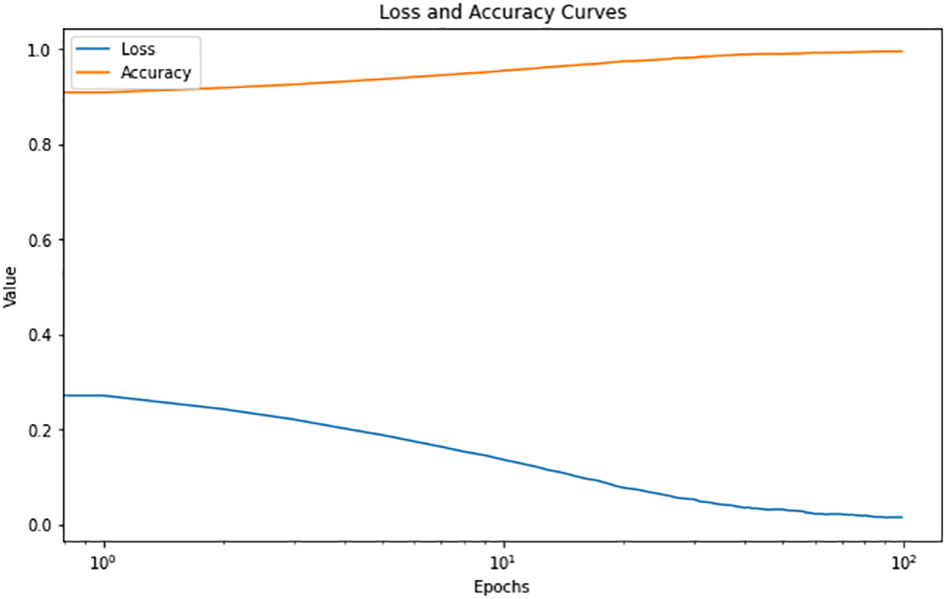
Model accuracy and loss changes during training.

The CNN model was trained using the training and validation data for 129 epochs. After this stage, the model ceased to show significant improvement, indicating that further training would not yield better results. In other words, the changes in weights were not meaningful, and further focus on training the model would not yield better results. **Figure 8** depicts the variation of the proposed model’s accuracy and loss values on the training data. Additionally, this figure showcases a visual representation of the model’s performance.

**Figure 9:**
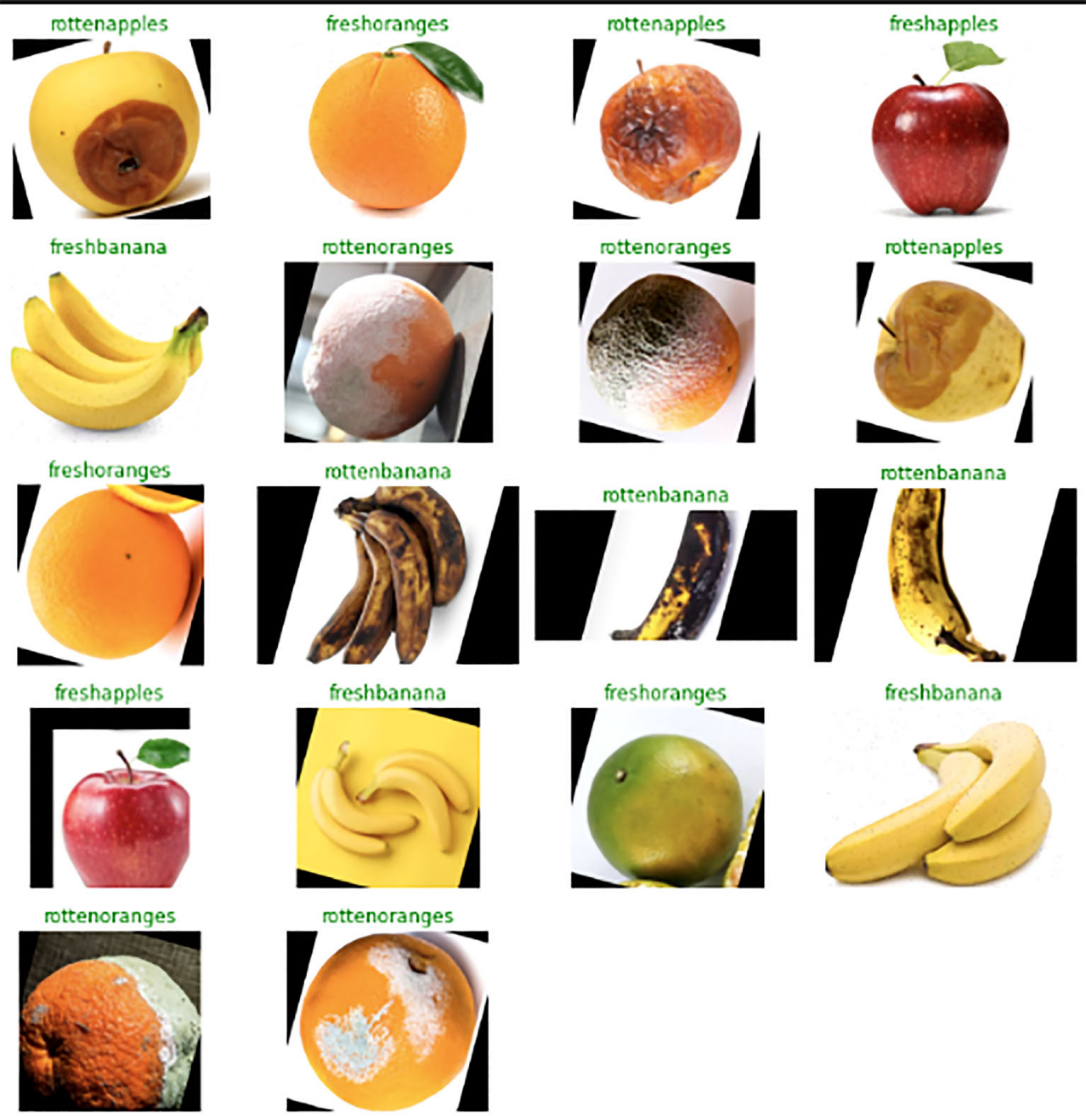
Model performance results after evaluation on test data.

To prevent overfitting, the early stopping callback is used. This callback evaluates the model’s performance in each epoch and stops the training process earlier if no improvement is observed. This decision ensures that the model, considering the information learned in previous epochs, is selected and saved. This version is considered the final result of the training and can be utilized with high accuracy for detecting fresh and rotten fruits. Based on the usage of this callback, the training of the model was stopped at epoch 129. Additionally, the model’s best performance was saved using model checkpoint, with the model from epoch 99 being considered the best performance. **Figure 9** showcases the performance of the proposed method on a selection of samples from different classes.

**Figure 10:**
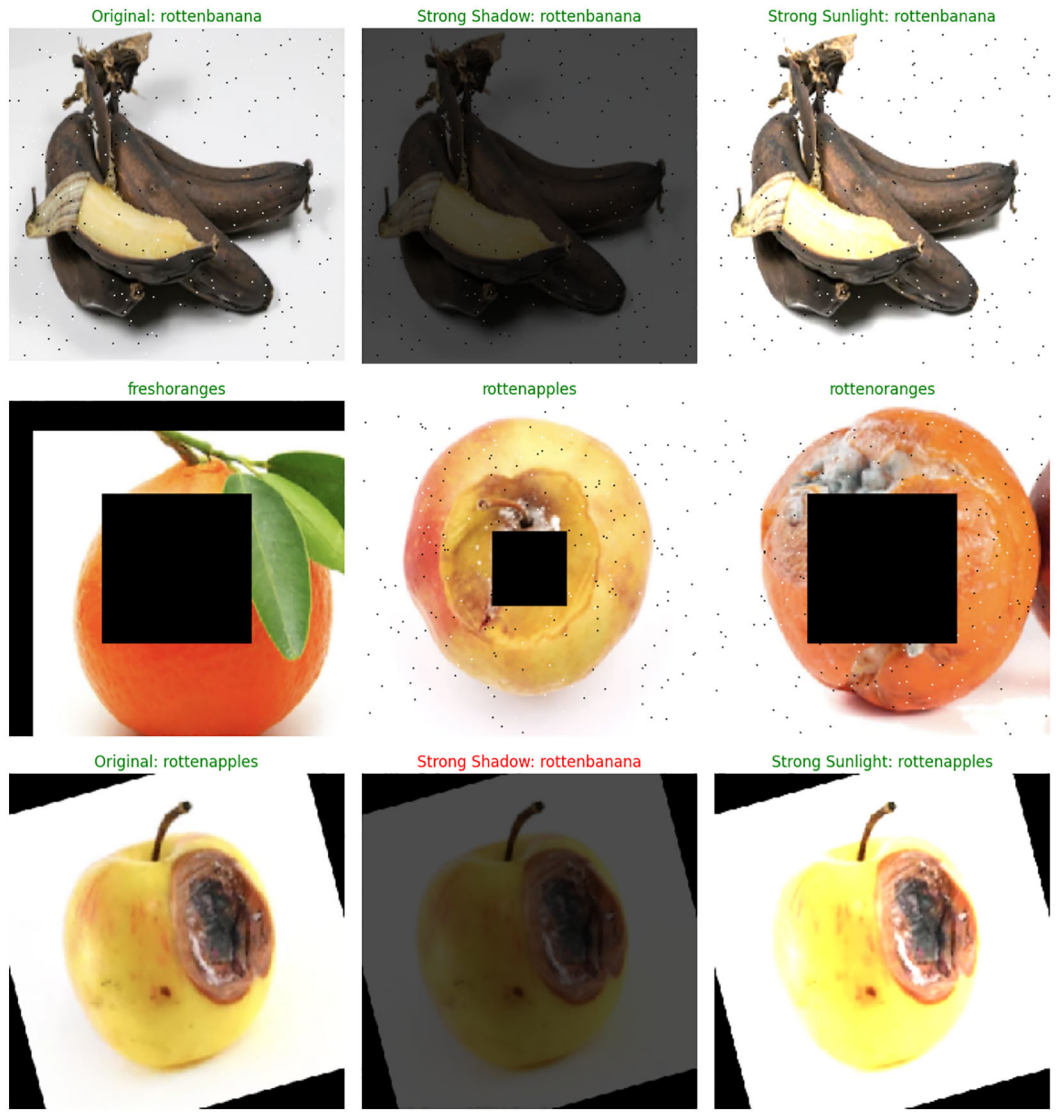
Model performance result for inputs with different conditions.

As **Figure 10** shows, this model underwent rigorous testing on datasets featuring challenging conditions, including intense shadows, extreme lighting variations, and partial image obstructions, yielding accurate results in most instances. Notably, under normal conditions, predictions were consistently accurate, showcasing the robust performance of the model. Only in cases with extreme shadows did the model occasionally exhibit errors. The comprehensive test results, encompassing predictions under both normal and challenging conditions, are depicted in **Figure 10**.

**Figure 11:**
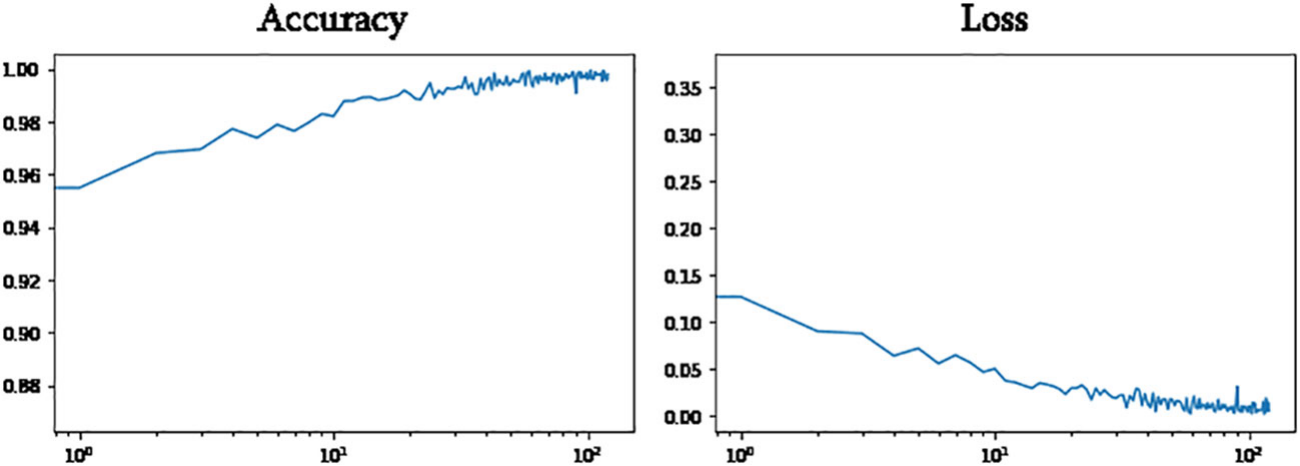
VGG16 model accuracy and loss changes during training.

**Figure 12:**
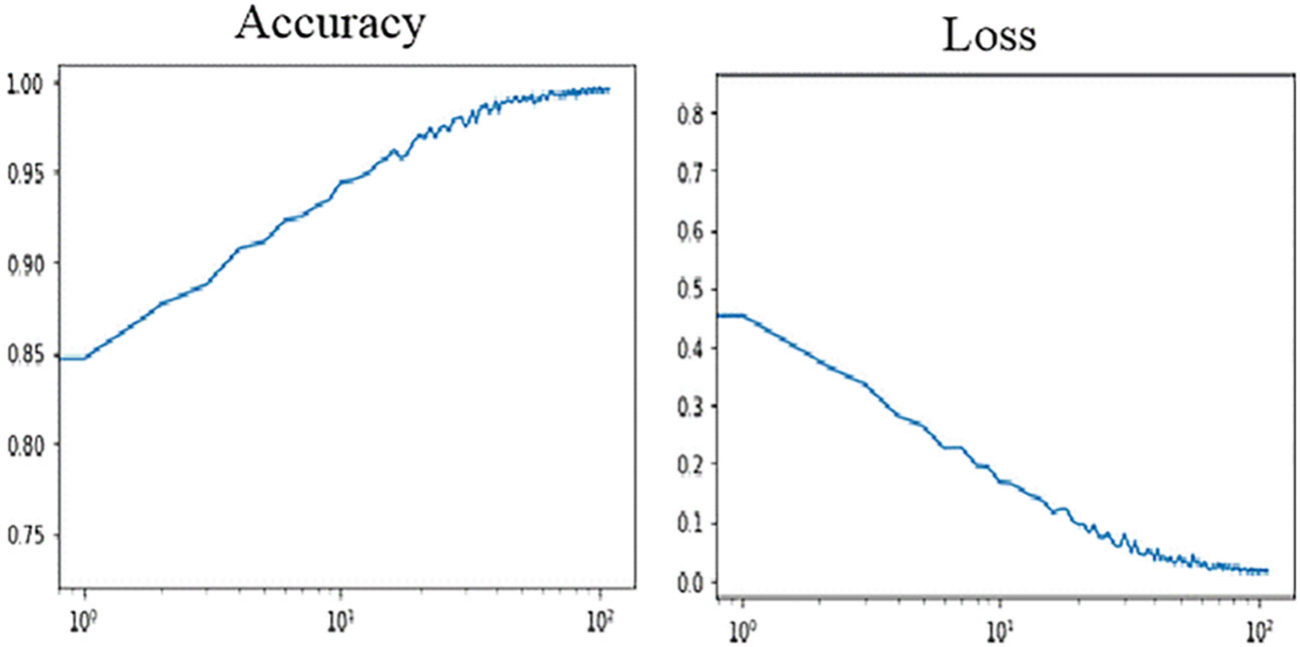
The proposed model accuracy and loss changes during training.

**Figure 11** shows, VGG16 model accuracy and loss changes during training. As shown in **Figure 12**, the proposed model achieved a training accuracy of 99.8% and a validation accuracy of 99.7%. The model achieved a test accuracy of 99.93%, demonstrating the model’s strong generalization capabilities. The libraries used for loading and processing images include Pandas, TensorFlow, Keras, PIL, and Matplotlib. A total of 2698 images were used to evaluate the model’s performance, and excellent results were obtained. These results indicate that the model accurately detects fresh and rotten fruits with high precision and can be used as a powerful tool in fruit-related industries. Based on the comparison depicted in **Figures 11** and **12**, it can be concluded that the proposed method outperforms VGG16. **Table 2** compares the proposed method with various state-of-the-art methods. This table presents a comparison between recent deep learning methods and other state-of-the-art approaches, allowing us to conclude that the proposed method outperforms these methods. This conclusion is based on the significant design aspects incorporated into the proposed network. The proposed network outperforms the “Transfer Learning with VGG16” and “Transfer Learning with Res-Net” approach as well as other networks such as VGG-16, LeNet-5, CNN, and AlexNet.

**Table 2:** Comparison of the accuracies.

Model	Accuracy
**VGG-16**	99.1
**VGG-19 (Pathak and Makwana, 2021)**	76.48
**LeNet-5 (Pathak and Makwana, 2021)**	82.93
**AlexNet (Pathak and Makwana, 2021)**	83.56
**CNN (Pathak and Makwana, 2021)**	98.23
**Proposed method**	99.93
**Transfer Learning with VGG16**	99.44
**Transfer Learning with Res-Net**	99.59

### 4.1 Confusion matrix

**Figure 13:**
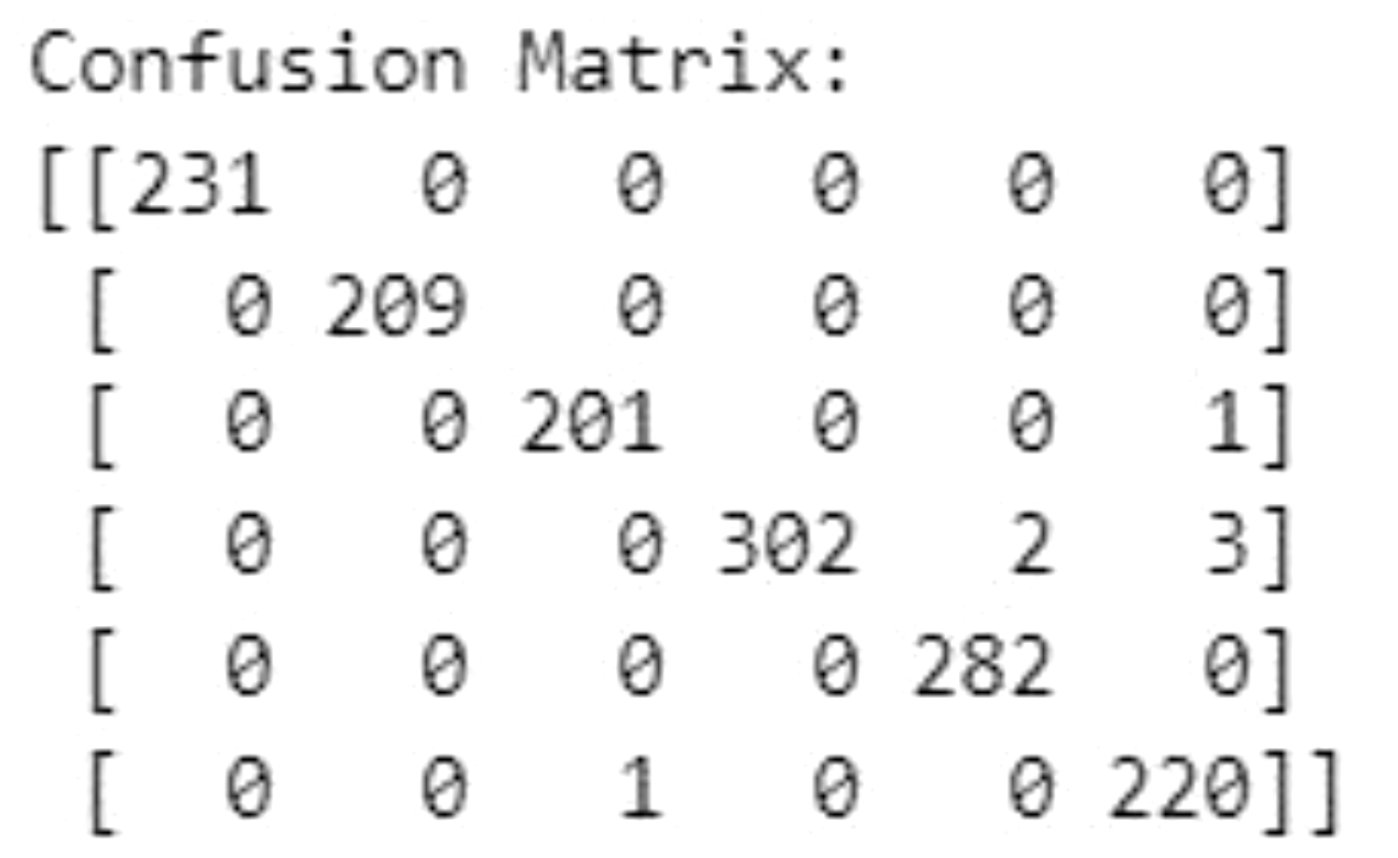
The confusion matrix.

The Confusion Matrix is a performance measurement for machine learning classification. It helps visualize the performance of an algorithm. As shown in **Figure 13**, the confusion matrix of the proposed model shows a high number of correct predictions, with only a few misclassifications, confirming the model’s robustness.

### 4.2 Class activation maps

To visualize influential regions in the decision-making process of our convolutional neural network (CNN), we employed Class Activation Maps (CAMs) using the GradCAM technique. CAMs provide a visual representation of critical areas within images that significantly contribute to accurate classification. These maps offer insights into the model’s attention by juxtaposing original images with CAMs, revealing impactful regions during classification.

**Figure 14:**
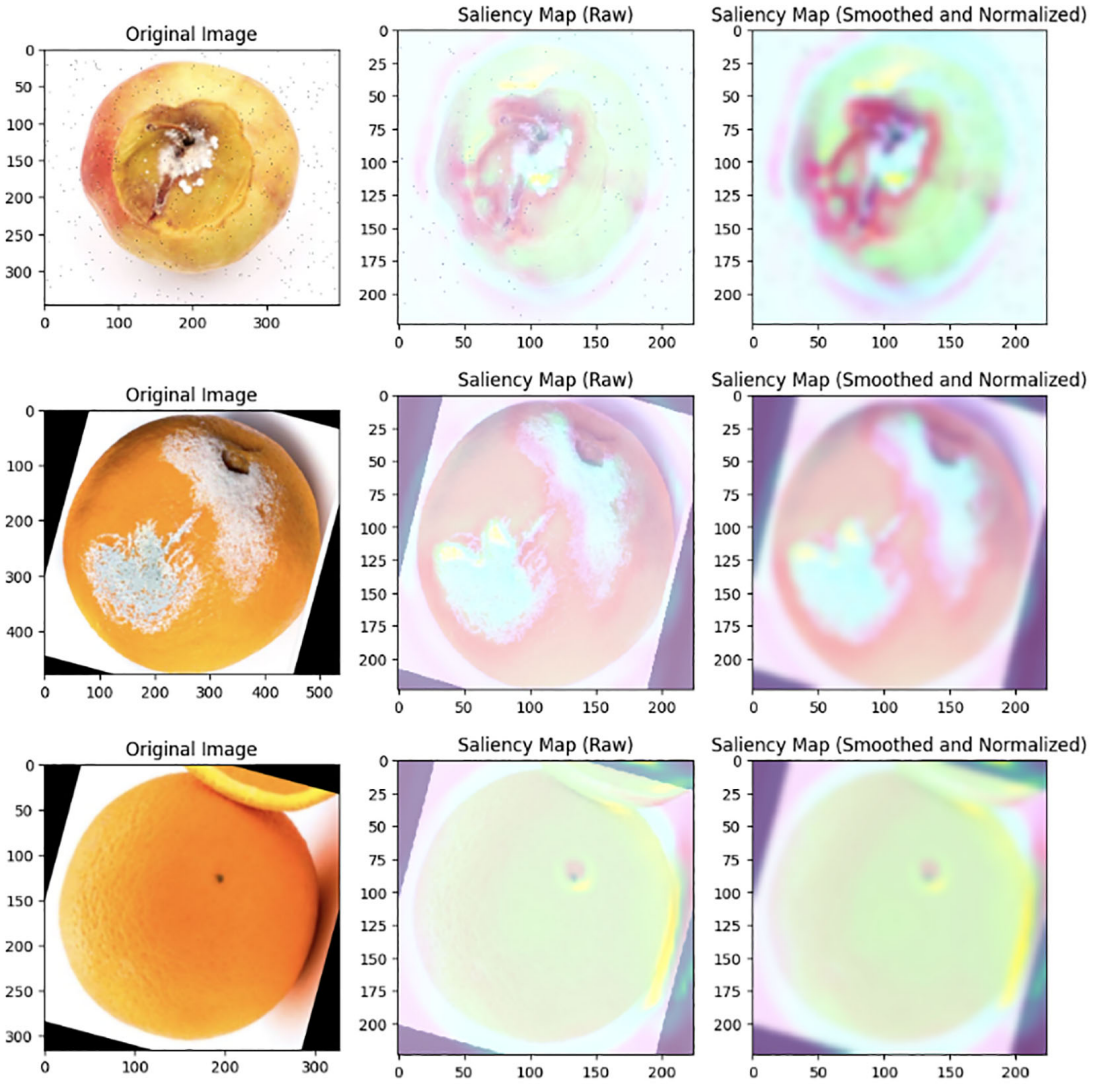
Insights from GradCAM illuminate key image features guiding CNN decisions.

By incorporating CAMs generated through GradCAM, we validate the alignment of the model’s focus with relevant image features, enhancing transparency in decision-making post-training. This visualization reinforces the efficacy of the CNN in recognizing and highlighting critical aspects of the input data. The performance and effectiveness of the model can be observed in **Figure 14**, where a selection of CAMs for specific images is presented.

## 5 Discussion

The successful implementation of an 11-layer convolutional neural network (CNN) for fruit quality control signifies a significant advancement in utilizing deep learning for agricultural purposes. The remarkable accuracy achieved during training, validation, and testing phases (99.8%, 99.9%, and 99.93% respectively) surpasses established methods, establishing this novel approach as a leading method for precise fruit classification.

Beyond its impressive accuracy, this methodology demonstrates more than just classification proficiency. It showcases robustness and adaptability, crucial for real-world applications, especially in domains where misclassification can have significant consequences. While existing models like VGG-16, VGG-19, LeNet-5, and AlexNet have proven effective in various domains, their application in fruit quality control reveals potential gaps in model generalization and customization, which the proposed model effectively addresses.

The meticulously crafted 11-layer CNN, optimized for the unique challenges of fruit classification, is not just a classifier but a result of strategic decisions. These decisions include thoughtful architectural design for nuanced feature extraction, as well as the implementation of data augmentation and early stopping techniques to mitigate overfitting and enhance generalization. This tailored approach enables the model to handle the diverse characteristics of different fruits, capturing intricate patterns and features that generic models may overlook.

The inclusion of Class Activation Maps (CAMs) not only enhances transparency but also facilitates continuous model refinement. By providing visual insights into decision-making processes, CAMs enable a deeper understanding and optimization of feature extraction and classification, leading to incremental improvements in model performance. The robustness of the proposed method is evident from the analysis of the confusion matrix, which reveals a high number of correct predictions with minimal misclassifications. This robustness reinforces the method’s reliability and its potential to be deployed in real-world fruit quality control scenarios.

In this section of the article, it is essential to note that due to constraints in the dataset in this domain, enhancing the model’s accuracy for various conditions, especially intense shadows, can be achieved with an increased dataset. Expanding the dataset for different scenarios, including intense shadow conditions, holds the potential to further improve the model’s performance.

Moreover, the model demonstrates versatility beyond fruit classification, extending its capabilities to identify various plant species. This broadens its applicability in diverse agricultural scenarios. Additionally, the model proves effective even with limited training data, making it a practical tool for deployment in different agricultural contexts.

The proposed model for automated agriculture systems in fruit protection introduces several significant advancements compared to previous approaches. These include robustness and generalization through handling variations in decay patterns and environmental conditions, effective handling of limited data scenarios using active learning, and data augmentation techniques, and a strong emphasis on interpretability and explainability through feature visualization. These advances enhance the practicality and performance of the proposed model, making it a novel and comprehensive solution for automated agriculture systems in fruit protection.

## 6 Conclusion

The proposed method has made significant strides in fruit quality control and other agricultural applications. Its custom model architecture, robustness-enhancing strategies, and versatility set it apart. While its exceptional accuracy sets a new benchmark, the holistic approach to design and application is what truly distinguishes it. It goes beyond being just a classifier, showcasing the integration of deep learning into specialized domains. The use of Class Activation Maps (CAMs) and a focus on transparency and model refinement are notable features. They improve decision-making and enable continuous model improvement through visual data and practical applications. This research has practical implications, particularly in enhancing fruit quality control and reducing economic waste. The model’s effectiveness in distinguishing between fresh and rotten fruits, as well as its robust performance validated through confusion matrices and CAMs, demonstrates potential beyond its current application. It can be extended to create accurate models for detecting plant-related videos, identifying species, monitoring growth, and detecting diseases. This broadens its applicability from industry to research. Sharing knowledge through web applications and software libraries can be a valuable resource across various fields, including agriculture and artificial intelligence.

## 7 Future work

In terms of future work, there are several areas that can be explored to enhance the proposed method. Firstly, data expansion through augmenting the dataset to encompass diverse conditions, including challenging scenarios like intense shadows, can significantly improve the model’s real-world accuracy. This would involve collecting and incorporating more varied and representative data to ensure the model’s robustness. Secondly, further refinement of the model’s architecture is essential. Through iterative exploration and fine-tuning, the adaptability and performance of the model can be enhanced across different conditions. This may involve experimenting with different network architectures, optimizing hyperparameters, and incorporating advanced techniques such as attention mechanisms or transfer learning.

Additionally, continuous evaluation and benchmarking against contemporary models will be crucial to ensure that the proposed approach remains at the forefront of accuracy and efficiency. Regularly assessing its performance and comparing it with state-of-the-art methods will help identify areas for improvement and guide future research directions. Furthermore, we can provide accessible resources for practical implementation in fields like agriculture and artificial intelligence. This would involve creating intuitive interfaces that allow users to apply the model easily and obtain valuable insights from the fruit decay detection and plant identification system. Overall, these future directions, including data expansion, model architecture refinement, technique exploration, and continuous evaluation, can contribute to advancing the proposed method and its potential impact in various industries and research fields.

## Data availability statement

Publicly available datasets were analyzed in this study. This data can be found here: https://www.kaggle.com/datasets/sriramr/fruits-fresh-and-rotten-for-classification. Three original packages have been used and/or developed in the framework of this study. All of these are publicly available on official repository and/or main DevOps platforms: https://github.com/pariyaaf/FruitDiseaseDetection-pariya.

## Author contributions

PA: Data curation, Formal analysis, Methodology, Software, Writing – original draft. TZ: Software, Supervision, Visualization, Writing – review & editing. MD: Project administration, Resources, Validation, Visualization, Writing – review & editing. MZ: Methodology, Software, Validation, Writing – review & editing.
