# Supplementary material for: Robust deep learning method for fruit decay detection and plant identification: enhancing food security and quality control
Source: Front Plant Sci. 2024 May 7;15:1366395. doi: 10.3389/fpls.2024.1366395 (PMC11106415; doi:10.3389/fpls.2024.1366395)
Supplement: Supplementary file 1 [file DataSheet_1.pdf]

Robust Deep Learning Method for Fruit Decay Detection and Plant Identification: Enhancing Food Security and Quality Control

Robust deep learning method detects fruit decay, highlights robustness, achieves 99.93% accuracy, and serves fruit industry with precise adaptation.

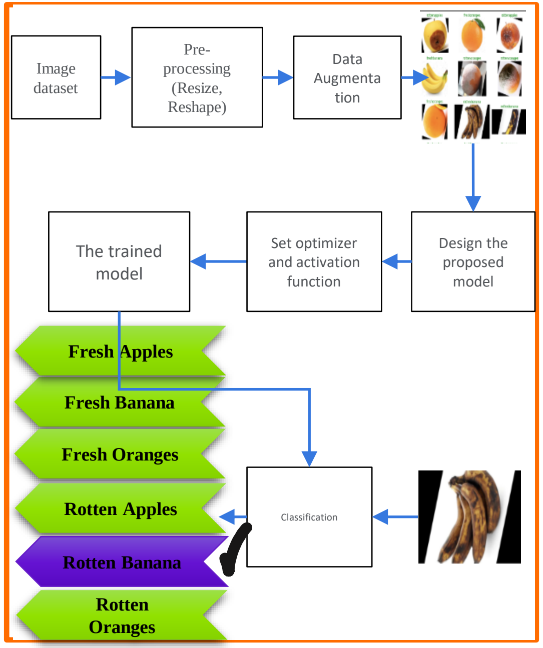

Block diagram of the proposed method

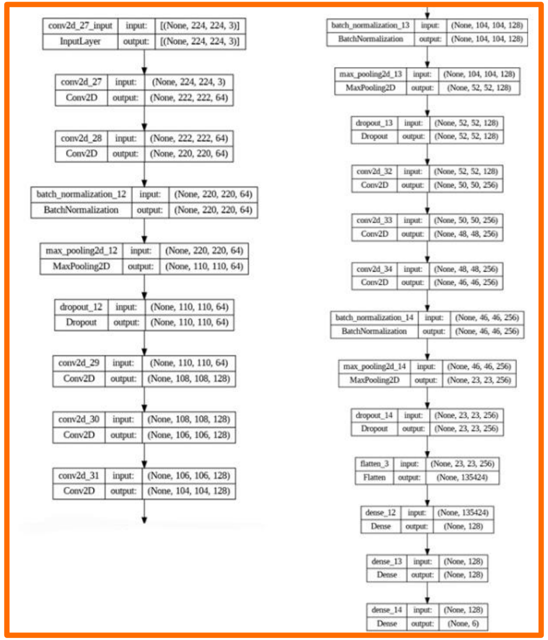

The proposed model structure

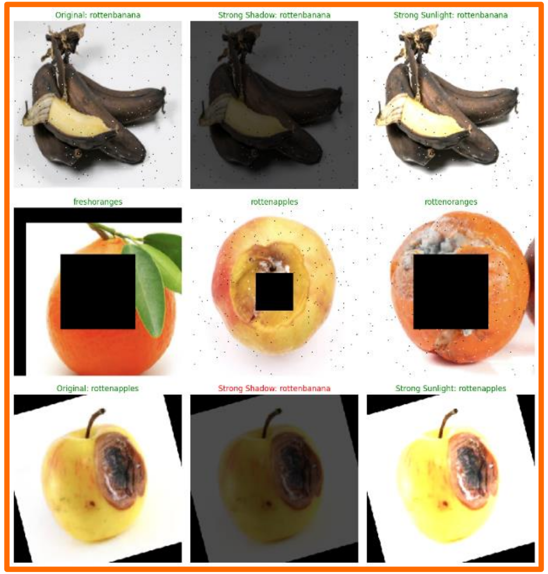

Model performance result for inputs with different conditions results
